# A Characterization of Internet Dating Network Structures among Nordic Men Who Have Sex with Men

**DOI:** 10.1371/journal.pone.0039717

**Published:** 2012-07-13

**Authors:** Antonella Villani, Arnoldo Frigessi, Fredrik Liljeros, Monica K. Nordvik, Birgitte Freiesleben de Blasio

**Affiliations:** 1 Department of Mathematics, University of L’Aquila, L’Aquila, Italy; 2 Department of Biostatistics, Institute of Basic Medical Sciences, University of Oslo, Oslo, Norway; 3 Department of Sociology, Stockholm University, Stockholm, Sweden; 4 Institute for Future Studies, Stockholm, Sweden; 5 Department of Social Work, Mid Sweden University, Östersund, Sweden; 6 Department of Infectious Disease Epidemiology, Division of Infectious Disease Control, Norwegian Institute of Public Health, Oslo, Norway; INSERM & Universite Pierre et Marie Curie, France

## Abstract

**Background:**

The Internet has become an important venue for seeking sexual partners and may facilitate transmission of sexually transmitted infections.

**Methods:**

We examined a 64-day data log of flirt messages expressing sexual interest among MSM within the *Qruiser.com* community. We used logistic regression to analyze characteristics of MSM sending and receiving flirt messages and negative binomial regression to examine individual activity and popularity. The structural properties, including the core structure of the flirt network, were analyzed.

**Results:**

The MSM population consisted of approximately 40% homosexuals and 37% bisexuals, while the remaining 23% included men who identified as heterosexual but searched for sex with men and “experimental”. MSM were more likely to send flirt messages if they were homosexual and aged 40+ years; young people aged < 30 years were more likely to receive a flirt. Possession of a webcam was strongly associated with both sending flirt messages and being a flirt target. The distributions of flirts sent (max k_out_ = 2162) and received (max *k_in_* = 84) were highly heterogeneous. Members in central cores were more likely homosexuals, singles, and aged 31–40 years. The probability of a matched flirt (flirt returned from target) increased from 1% in the outer core to 18% in the central core (core size = 4).

**Discussion:**

The flirt network showed high degree heterogeneity similar to the structural properties of real sexual contact networks with a single central core. Further studies are needed to explore use of webcam for Internet dating.

## Introduction

In recent years, the internet has become a major driver for social interactions and has made it possible for people to communicate quickly and over long distances. Alongside this development, internet dating has become increasingly popular and a socially acceptable way to meet partners for dates and relationships [1]. In 2000 an outbreak of syphilis was traced linked to men who have sex with men (MSM) chat room participants [2], and this has fueled speculation that the internet may function as a risk-promoting environment for spread of sexually transmitted infections [3]. However, recent findings seem to suggest that seeking sexual partners online is a marker of high-risk sexual behavior, whereas meeting online partners does not in itself promote high-risk behaviors [4], [5].

Online dating is different from traditional ways of meeting sex partners, e.g. frequenting bars, due to the larger user base, the greater anonymity and flexible self-presentation options [6], and dating people need not be online at the same time. Furthermore, the internet has the ability to transcend social and geographic barriers, which may have an impact on sexual contact networks, and influence disease transmission [7].

Studies of sexual contact networks show a large variation in numbers of contacts [8] and indicate that the structural properties of sexual contact networks are crucial to the spread of STIs as highly sexually active individuals are responsible for a disproportionate number of transmission events. Highly sexually active individuals are more likely to become infected, and when infected they can spread infection more effectively. The spread of infection can be further enhanced if individuals with many contacts most frequently mix with each other (assortative mixing) [9]. Such individuals with high turnover rates of partners and a high internal contact may form one or several cores separated by less dense regions in the network which may permit persistence and spread of STIs which are not able to reproduce themselves in the general population. A study on network models for STI transmission suggests that network centrality is mainly associated with risk of acquiring infection, while the number of contacts has the largest influence on the risk of transmitting infection [10]. A *k*-core analysis can be conducted to evaluate if a network has a core-periphery organization, with central and densely connected cores structures surrounded by peripheral and less cohesive units [11]. In this method, the network is split into clusters, where member of the *k*-core are tied to at least *k* numbers within their cluster. The network core centrality identifies key members as those with the highest degree of clustering. The cores may be isolated from each other by less dense regions in the network. The existence of several separated core groups in a population may reflect geographical regions, or indicate that there are different types of high-risk groups who may need different types of intervention. Multiple core-groups may further allow for different strains of a STI to circulate simultaneously in a population, and make it more difficult to eradicate an STI, as the various core-groups can act as reservoirs for each other.

The aim of the present paper was to study online dating patterns among men who have sex with men (MSM) and to compare the results with findings from previous research on real sexual networks. First, we analyzed the demographic profile of active MSM members and their partner preferences. Second, we studied the structural properties of the flirt network, and we explored the core structures to identify if highly active flirters were tightly connected or scattered over the network. Finally, we analyzed which characteristics of the members were more (or less) pronounced when moving from the periphery to the center of the communication network.

## Methods

### Data

The data set consists of a complete log of anonymized communications on Qruiser.com between 16∶25 on November 15, 2005 and 10∶08 on January 18, 2006, corresponding to approximately 64 days. Qruiser.com is intended for homosexuals, bisexuals and experimental men and women and had approximately 109,000 members at the time of the study. Members of the community are named by an alias, and have their own homepage, where they may describe themselves in terms of age, profession, cohabiting and marital status, looks, sexual identity, sexual style and the type of partners or friends they are looking for. Personal information is viewable by other members.

There are three major ways to communicate in the community: *Messages*, where members exchange private messages, similar to emails, *Guest books*, where members post open messages on other members’ homepages, and *Flirts,* where members can express specific sexual intention/interest. The identity of the member posting a flirt is not disclosed to the target, unless the target returns the flirt within a time period of one month. In this case, the pair is notified that they have a matched flirt and the identity of the other member is revealed.

### Ethics Statement

De-identified data from the Qruiser community was collected in 2006 after being approved by the Regional Ethical Review Board in Stockholm, record 2005/5∶3. In 2006, QX Publishing Ltd. who stores the data did not have a privacy policy and there were no clause on data sharing with third parties. However, today the company has changed its policy to include a confidentiality statement, which prevents further data sharing. QX Publishing Ltd. received payment from us to cover costs associated with data extraction only. Re-identification of individuals is not possible because the demographic information was restricted to county of residence (in Swedish: län); Sweden is divided into 21 counties. Member names, Qruiser aliases, physical addresses or IP addresses were not accessible, effectively preventing person identification. According to the contract with QX Publishing Ltd., the data set can only be made available to control the accuracy of the analyses conducted in this study (and provided that this can be done in such a way that data is not stored outside of our control).

### Study Population

The *MSM population* was formed by selecting all male Nordic members with a stated preference for sexual contact and with a stated preference for male contacts, including men with a stated preference for men and women (N = 19,549). The remaining Nordic population is referred to here as the *non-MSM population* (N = 85,220). This restrictive selection was made to facilitate a clear interpretation of results.

### Statistical Analyses

The power law scaling exponents of the degree distributions were fitted adopting a maximum likelihood estimation procedure [12]. The degree-degree correlation was estimated by the Pearson correlation coefficient [9].

We used logistic regression to identify the characteristics of MSM members sending flirt messages to other MSM members (*proposers*). Similar analyses were conducted to characterize MSM members receiving flirts from other MSM members (*targets*), compared to MSM members who did not receive any flirts or who received flirts exclusively from outside the MSM group. The following variables were included in the analyses: 1) transsexual identity, 2) sexual orientation (homosexual; bisexual; experimental), 3) single civil status, 4) age (<30 years; 30–39years; 40+ years), 5) age preference of partner (older or equal age; younger or equal age; same age; no preference, including no stated preference), 6) webcam. The variable experimental included people with a self-reported sexual orientation of being experimental, heterosexual, queer, asexual and people without a stated preference; these groups accounted for 5.5% of the MSM population (Table 1). A stepwise backward variable selection method was employed using a significance level of p<0.05. We used a negative binomial regression to characterize the popularity and activity (in terms of the numbers of flirts received or sent) of the MSM members to account for over-dispersion. The variables in these analyses were identical to the variables used in the logistic regression described above.

**Table 1:** Demographics of the Qruiser population (November 2005 –January 2006).

	MSM population	Nordic population
	(N = 19,549)	(N = 104,768)
	No.(%)	No.(%)
**Country**		
Sweden	17,307 (88.5)	97,535 (93.1)
Norway	1,221 (6.3)	4,328 (4.1)
Denmark	601 (3.1)	1,510 (1.4)
Finland	414 (2.1)	1,396 (1.3)
**Gender**		
women	–	35,618 (34.0)
men	19,549 (100)	66,089 (63.1)
other	–	3,062 (2,9)
**Age**		
Mean years	36.16 (std 11.76)	32.17 (std 11.32)
≤30 years	7,046 (36.0)	54,893 (52.4)
31–40 years	6,136 (31.5)	28,977 (27.7)
41+ years	6,336 (32.5)	20,899 (19.9)
**Sexual orientation**		
homosexual	7,767 (39.7)	29,315 (28.0)
bisexual	7,190 (36.8)	27,067 (25.8)
experimental	3,510 (18.0)	16,090 (15.4)
queer	130 (0.7)	2,112 (2.0)
heterosexual	294 (1.5)	20,410 (19.5)
not stated (incl. Asexual)	642 (3.3)	9,168 (8.7)
**Transsexual**	359 (1.8)	2,436 (2.3)
**Civil status**		
single	10,971 (56.1)	42,192 (40.3)
have partner	1,072 (5.5)	10,271 (9.8)
have several partners	513 (2.6)	1,839 (1.8)
married /live with partner	2,095 (10.7)	10,871 (10.3)
not stated	4,898 (25.1)	39,596 (37.8)
**Looking for**		
Sex	19,549 (100)	35,804 (34.2)
Other	–	31,474 (30.0)
not stated	–	37,490 (35.8)
**Have webcam**	2,079 (10.6)	8,849 (8.4)

Analyses were conducted using the software *Matlab R8* and *Stata 7.0*; the core analyses were made with *Pajek and MathCad 14*.

## Results

### Demographic Characteristics of the MSM Members

The MSM population comprised 18.6% of all Nordic members. The vast majority of MSM members (88.5%) were Swedes, and the mean age of the group was around 36 years. Among the MSM, 39.7% identified themselves as homosexuals, while 36.8% and 18.0% stated their sexuality as bisexual and experimental, respectively. There were 56.1% singles, while 25.1% did not state their civil status. Approximately 1 in 10 of MSM members had a webcam.

During the study period, 16% (3,044/19,549) of the MSM members sent at least one flirt, and 33% (6,510/19,549) of the members received at least one flirt.

### Characteristics of MSM Members Sending and Receiving Flirt Messages

Table 2 presents results of a logistic regression examining the characteristics of MSM proposers, who sent flirt messages to other MSM members. The reference category was MSM members who either did not send any flirt messages, or who flirted exclusively with members outside the MSM group. Because of the large sample size, we mainly address significant results using a P value of 0.001. We found that MSM members with a stated homo- or bisexual orientation, aged 40+years, and members looking for a younger partner were more likely to be a proposer (i.e. a person sending flirts). Table 3 shows the characteristics of active MSM proposers based on a negative binomial regression; this analysis was restricted to members who had sent at least one flirt to a MSM target. Sending many flirts was associated with being single, older than 40 years, and a preference for younger partners; transsexuals were less likely to send many flirts. Sexual orientation was not significant for explaining flirt activity. A strong association was found between having a webcam and being both proposer and highly active.

**Table 2:** Results of logistic regression analysis for demographic characteristics of MSM proposers (N = 19,549).

	Odds ratio	95% CI	p-value
Partner preference			
Younger	1.57	1.26–1.96	<0.001
Older	1.31	1.02–1.67	0.03
No preference	1.35	1.08–1.68	0.01
Age			
<30 years	0.59	0.52–0.67	<0.001
30–39 years	0.74	0.65–0.84	<0.001
Sexual orientation			
Bisexual	1.20	1.03–1.41	0.02
Homosexual	1.74	1.50–2.02	<0.001
Webcam	2.10	1.84–2.41	<0.001

Only significant covariates are shown. An odds ratio above/below 1 indicates that this characteristics was more/less present among MSM proposers sending flirts to other MSM members, compared to MSM members who either did not send flirt messages, or MSM members who sent one or more flirt messages to non-MSM members only. A likelihood ratio test of the full model with respect to the pure intercept -2LL = 299.35 (p-value<0.00001).

**Table 3:** Results of a Negative Binomial regression analysis of the number of flirts sent by MSM proposers^1^ (N = 1,592).

	Coefficient	95% CI	p-value
(overdispersion)	3.72		<0.001
Partner preference			
Younger	0.34	0.03–0.67	0.03
Age			
<30 years	−0.51	−0.70 – −0.25	<0.001
40+ years	0.43	0.19–0.69	<0.001
Sexual orientation			
Transsexual	−1.08	−1.87 – −0.28	0.01
Civil status			
Single	0.32	0.11–0.53	<0.001
Webcam	0.59	0.32–0.85	<0.001

1 Only MSM proposers who sent at least one flirt to a MSM target were included.

Only significant covariates are shown. A positive/negative sign of a coefficient indicates increasing/decreasing activity (in terms of numbers of flirts sent) among MSM members with this characteristic. Likelihood ratio test against the constant model was -2LL = 91.90 (p-value <0.0001). The overdispersion parameter was estimated at 3.72 suggesting that the negative binomial model fitted the data better than a Poisson one.

Similar analyses were conducted to identify characteristics of MSM targets, who received flirt messages from other MSM members, from those who did not receive any flirts, or who were targets of non-MSM members exclusively. We found that MSM members with a stated homosexual or bisexual identity, aged < 30 years, and members preferring older partners were more likely to be a target (Table 4). The popularity of the MSM members, measured in terms of the number of flirt messages they received, was higher in members with a stated homosexual identity, singles, and members aged < 40 years (Table 5). This analysis was limited to MSM members who received at least one flirt from within the MSM group. Again, we observed a strong association between having a webcam and being a MSM target.

**Table 4:** Results of logistic regression analysis for demographic characteristics of MSM targets^1^ (N = 19,549).

	Odds ratio	95% CI	p-value
Partner preference			
Younger	1.18	1.02–1.36	0.02
Older	1.88	1.62–2.19	<0.001
No preference	1.66	1.44–1.90	<0.001
Age			
<30 years	1.27	1.17–1.38	<0.001
Sexual orientation			
Bisexual	1.26	1.14–1.38	<0.001
Homosexual	1.73	1.58–1.90	<0.001
Webcam	1.86	1.68–2.05	<0.001

Only significant covariates are shown. Likelihood ratio test of the full model with respect to the pure intercept -2LL = 451.82 (p-value <0.00001).

**Table 5:** Results of a Negative Binomial regression analysis of the number of flirts received by MSM targets (N = 4,550).

	Coefficient	95% CI	p-value
(overdispersion)	1.48		<0.001
Partner preference			
No preference	0.09	−0.01–0.20	0.08
Age			
<30 years	0.48	0.38–0.58	<0.001
30–39 years	0.50	0.39–0.60	<0.001
Sexual orientation			
Homosexual	0.35	0.26–0.44	<0.001
Civil status			
Single	0.15	0.07–0.23	<0.001
Webcam	0.27	0.16–0.37	<0.001

1 Only MSM victims who received at least one flirt from a MSM target were included.

Five significant covariates (plus one boarder line) are shown. Likelihood ratio test against the constant model was -2LL = 237.40 (p-value <0.0001). The overdispersion parameter was estimated at 1.48 suggesting that the negative binomial model fitted the data better than a Poisson one.

The probability for a MSM member to be both sending and receiving flirts was greater among homosexuals OR = 1.43 (95% CI 1.19–1.71), members preferring younger partners OR = 1.34 (1.05–1.71), and people owning a webcam OR = 1.47 (1.20–1.79). In Table S1 and Table S2 we present further results showing the similarities between flirt proposers and targets. In particular, we found no strong tendency for MSM members to flirt within their own age group. Nevertheless, 50% of proposers in the 20–30 year age group preferred to flirt with a member of the same age (Table S2).

### Structural Properties of the Flirt Networks

**Figure 1:**
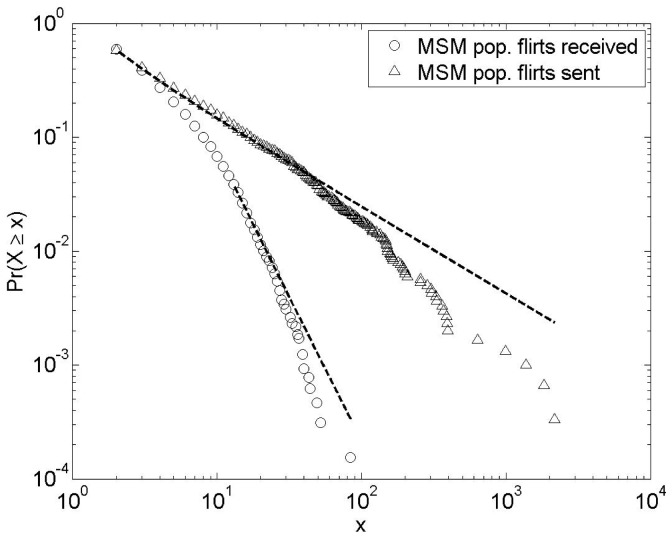
Cumulative degree distribution of the flirt messages received (N = 21,687;circles) and flirt messages sent (N = 33,865 ;triangles) by the MSM population during the study period. *Lines indicate the best fitting power law model.*

The flirt message network is a directed network where the connectivity of each member is described by 2 values: the outgoing connectivity (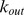) and the incoming connectivity (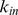). The cumulative degree distributions 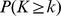 of flirts sent and received by MSM members were both highly right-skewed (Fig. 1). The highest variability was observed in the number of outgoing flirts with a maximum of 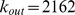, whereas the largest number of flirts received by one member was 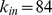. We found no support for a power law degree distribution in either the out-going or in-going flirt network; cf. Fig. 1, legend text.

Table 6 shows the Pearson degree correlation coefficient 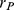 for the flirt message network composed of flirts exchanged between MSM members (MSM-MSM), and the network composed of flirts sent by MSM members to all Nordic members (MSM-Nordic). A network where high- or low- connectivity vertices preferentially connect to vertices with similar connectivity is called “assortative” (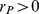), while a network where vertices have a tendency to attach vertices of different connectivity is named “disassortative” (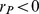). When neglecting the direction of the flirts, the network was found to be weakly disassortative, as seen from the overall negative 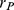 values (first column). We found assortative mixing for 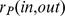 and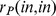, suggesting that both active MSM flirters (large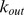) and popular MSM members (large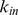) had a tendency to flirt with popular MSM members. We repeated the analysis on networks, where multiple flirts were collapsed, i.e. only a single connection between a proposer and a target was counted (Table 6; bottom section). In this analysis, active MSM members were found to connect in a disassortative way to popular MSM members, while no significant correlation was obtained in the flirts exchanged between popular MSM members. Together these findings suggest that active MSM members had a tendency to flirt many times with few selected members, thereby making them popular, while in general active MSM members had a tendency to select different targets. Overall, the corresponding networks including Nordic targets had similar characteristics.

**Table 6:** Pearson correlation coefficients.

		(proposer; target)			
	Non-directional	(out;out)	(out;in)	(in;out)	(out;out)
**proposer-target** **(#edges)**					
Multiple connections					
MSM-MSM(11,379)	−0.141	−0.033	−0.019	0.190	0.184
MSM-Nordic (33,865)	−0.157	−0.019	−0.006	0.138	0.136
Single connections					
MSM-MSM (7,763)	−0.162	−0.035	−0.017	−0.073	−0.023
MSM-Nordic (24,231)	−0.153	−0.020	−0.004	−0.101	−0.011

### The K-core Structures

**Figure 2:**
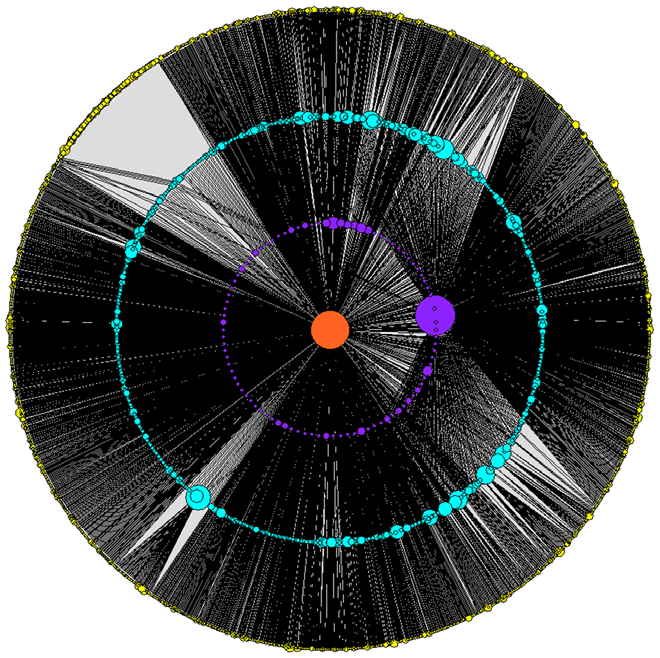
The core structure of the MSM exclusive network. *The numbers of individuals in the different cores were: 1-core: 3261; 2-core: 1302; 3-core: 564 and 4-core: 249. Each node in the graph represents an interconnected sub-cluster of nodes with similar core number. The sizes of the nodes represent the logarithmic size of the clusters.*

We performed a core analysis to identify subsets of the network, named *k*-cores, of increasing inter-connectedness (centrality). In these analyses we collapsed the directed networks of outgoing and ingoing flirts to a non-directed network. The *k*-cores are found based on a recursive thinning strategy: at each step, all members with only one flirt contact in the network are removed; the procedure is repeated until there are no members left. Members removed in the first thinning were assigned with a *k*-value of 1; members removed in the second thinning procedure were assigned with a *k*-value of 2, and so on. The last core represents the center of the network. The MSM flirt network had a core size of 4. A weakness with the core analysis is that it fails to differentiate between networks where all nodes with the highest core number belongs to the same core, and those cases where there are multiple cores separated by of less dense regions of the network. To overcome this problem, as well as reducing the number of nodes in the graph we show a collapsed graph (Fig. 2.) of the MSM flirt network in which members with a similar *k-*value who are reachable through links between members in the same shell have been collapsed into one node. The size of the nodes represents the log of the number of members in the collapsed nodes. The graph shows that there is just one large central core in the network. Interestingly, individuals outside the central core (*k<4)* are not homogeneously distributed, but are organized in a series of isolated clusters in the sense that clusters with similar core number only can reach each other through a path that either goes through individuals with higher core numbers (usually members of the central core) or more rarely through individuals with lower core numbers. We performed a linear regression to identify characteristics of MSM members in the central network compared to members in the periphery of the network (Table 7). The analysis showed an association between homosexual identity, single civil status, age 31–40 years, possession of a webcam, and increasing core number. There was also a tendency for central MSM members to have a preference for younger partners.

**Table 7:** Results of linear regression of the member characteristics in the cores of the MSM flirt network and the Nordic flirt network.

	MSM → MSM (4 cores)			Nordic → Nordic (10 cores)		
	Coeff. (SE)10−2	Stand.coeff.B	Const.	Coeff. (SE)10−2	Stand. coeff.B	Const.
GenderManWomen	– –– –	– –– –	– –– –	4.0(0.1)−3.8(0.1)	0.17−0.16	0.560.42
Sexual orientationhomosexualbisexualother sexuality	7.2 (0.8)−4.4 (0.8)−2.7 (0.6)	0.12−0.08−0.06	0.350.420.23	3.8(0.1)−1.7(0.1)−2.0(0.1)	0.16−0.08−0.08	0.240.300.47
Civil statusSingleHave partnerNot stated	1.7 (0.8)−1.3 (0.6)−0.03 (0.7)	0.03−0.03−0.01	0.550.190.26	1.9(0.1)−1.8(0.1)−0.1(0.1)	0.08−0.09−0.01	0.410.250.34
Age≤30 years31–40 years41+ years	−2.2 (0.8)2.2 (0.7)−0.1 (0.7)	−0.040.040.00	0.410.270.32	0.4(0.1)0.5(0.1)−0.9(0.1)	0.020.02−0.05	0.530.250.24
Preference gender*ManWomenTransNot stated	– –−6.5(0.7)−3.6(0.6)– –	– –−0.12−0.09– –	– –0.400.21– –	2.4(0.1)−3.2(0.1)−0.7(0.1)−0.2(0.1)	0.10−0.13−0.05−0.01	0.470.480.100.30
Webcam	3.2 (0.6)	0.08	0.11	1.5(0.1)	0.09	0.10

* Not coded exclusive; member may want contact both with men and women; the sum of men, women, transsexual >100%.

We repeated the analysis for the Nordic network, which had a core size of 10. The results were consistent showing that high core numbers were associated with being male, homosexual, single, in possession of a webcam, and with preference for younger partners. People over 40 years were less likely to be in the center of the network as were people with a stated non-sexual contact preference.

### Characteristics of MSM with Matched Flirts

Only a minority of flirts was answered by the target member within the allowed one month period, whereby the members’ identities would have been disclosed (matched flirt). During the study period, we observed a total of 257 matched flirts involving at least one member of the MSM population (Fig. S1). Of the matched flirts, 20% were formed between two MSM members, while 80% involved a person from outside the MSM group. The success rate among the MSM proposers was 8.43% (254/3044), and 1.5% of the MSM proposers obtained more than one matched flirt (maximum 13). However, the observed success rate is likely underestimating the true value; data from roughly half of the study period was subject to either right- or left censoring (flirts could potentially be matched either before or after the study period).

Using logistic regression we found that homosexuals were more likely to have their flirts matched OR = 1.51 (95% CI 1.14–1.99), as were members in possession of a webcam OR = 1.47 (95% CI 1.04–1.90), whereas transsexual proposers were less likely to be successful OR = 0.56 (95% CI 0.35–0.89). No association between sending flirts to many different targets and higher numbers of matched flirts was found, suggesting that the most successful flirters were MSM members sending flirts to few selected targets, and these contacts could give rise to more than one matched flirt.

The probability for a MSM member to have at least one matched flirt increased almost linearly from 1% in the outer core to 18% in the inner core of the MSM-MSM network; the mean number of flirts sent and received also increased from 1.0 (median 0) in the periphery to 66.1 (median 5) in the inner core (Fig. S2).

## Discussion

We have analyzed the online dating structures among a group of MSM in order to study to what extent the network structure displays structural properties that may facilitate the spread of STIs. Sexual contact networks are reported to be highly skewed, but there is disagreement about whether they can be represented as power law networks [13], [14]. We observed a broad distribution in the number of contacts, but we found no support for power-law tails. The out-degree distribution was far more skewed than the in-degree distribution. The difference can probably be explained by the fact that an individual’s out-degree is the sum of one individual’s actions while the in-degree of an individual is the sum of many individuals’ actions. The large variation in individuals’ motivation to find new partners [11] will hence be reflected in the out-degree distribution but not in the in-degree distribution. The lower but still large variation in number of flirts received is more likely to reflect a difference in the attractiveness in the personal descriptions of the members on the site.

Our results show that the flirt network exhibit similar structures to real sexual contact networks. The large variation in contact numbers and core structure are features that can act to enhance spread of STIs and sustain transmission above the epidemic threshold, compared to a random network with a similar average mean value of contacts. We observed a single large central core surrounded by several heterogeneous sub-cores in the lower shells (Fig2); this finding may be of concern if it is representative of the true sexual network structure because one large central core will support infection transmission and probably increase the probability for non-core members to become infected. The observed organization of disjoint non-central cores that can reach each other only through the central core is likely to enhance importance of the core for epidemic process as well as its importance for potential strategic interventions. We further observed a weak disassortative interaction, and the scale of the flirts sent greatly exceeded the numbers of flirts received by any individual, indicating that while the internet poses great potential for finding partners, it also becomes increasingly difficult to get selected because of the great number of users. Sexual networks are different from many other types of networks as the contact numbers (i.e. popularity) are not visible and therefore cannot be used as a selection criterion. In the MSM exclusive network the characteristics most associated with increasing core number was homosexual identity, possession of a webcam, single civil status and people in the age group 31–40 years.

There was a tendency for central MSM members to have a preference for younger partners. We found that older men aged 40+ years were most active in the community, while younger men aged < 30 years were less active, which is in contrast with previous findings showing that an MSM engaged in internet dating is usually younger than traditionally recruited MSM samples [13], [14]. In addition, we found a preference among older men to search for younger partners, and the internet may facilitate contact across age group. Otherwise, our results show a general lack of assortativity by age in the flirt network. Both hetero- and homosexual men have similar age preferences for younger sex partners [15]. Though, partner age preferences have been shown to vary importantly depending on if a person is seeking in real life (IRL) long-term or short-term relationship: men seeking sexual contacts prefer significantly wider age range of partners compared to men looking for a steady partner [16]. Thus, lack of assorativity by age in the flirt network may suggest that the contacts are for sex and of a transient nature [17], [18]. It may also be that the flirts are intended for cybersex, where potentially there are no strong partner age preferences. However, to our knowledge, partner preferences of cybersex contacts are not well documented.

One interesting finding that needs to be addressed further in future research is the observed association between possession of a webcam and i) high flirt activity, ii) popularity and iii) high likelihood of having a matched flirt. Webcam can be used to view potential sex partners before meeting in real life. Another interpretation is that webcams are used for cybersex. A Swedish study concluded that almost 1 in 3 persons using internet dating websites engage in cybersex [19], and homo- and bisexual men and singles are more likely to have experienced cybersex compared to other groups [20]. It is notable that many MSM participating in the flirt network identify as heterosexuals. In a study of this group of men it was found that they agree to do things online that they would not do offline, and that they participate more often in cybersex compared to MSM who identify as homo- or bisexual [21]. Finally, it may also be that people involved in the flirt network use webcam for communication in general.

There are a number of limitations to this study. The interpretation of internet data is difficult because it is unknown whether the contacts actually lead to real sexual encounters. It is well documented that the internet has a disinhibiting effect on behavior [22], and the effort and time spend on sending a flirt is minimal compared to flirting with people in real life. In addition, flirts are anonymous unless returned within a specified time frame, and people are therefore generally not aware if they are being flirted with, which decrease the chance of flirts being matched. This strategy protects flirters from losing status if their efforts turn out non-successful, and may increase the likelihood for shy or uncertain members to engage in flirting compared to the face-to-face world. The flirt communication is embedded in the general information exchange on the site: each homepage contains a log of member aliases with time stamps showing who recently visited the homepage, and members may actively direct attention to themselves by posting messages, writing in guestbook, sending emails or declaring other members as favorites. For example, we found that only 0.5% of flirts were matched if no prior communication (favorite, guestbook, messages) were detected between proposer and target members in the study period, while this proportion increased almost 6-fold to 2.9% among flirt couples where previous interaction were found. If these potential behavioral differences in the online world are not consistent with the offline world through formation of sexual contacts, the flirt network is not a proper representation of real world high-risk sexual contact networks.

There were other limitations to the present study. First, a large proportion of Qruiser members (36%) did not state their preference for contact (sexual, friendship etc.), and these men were excluded from the MSM population in the analyses. Many of these members are likely seeking sexual partners, but have chosen a higher level of anonymity. Indeed, the majority of flirts (66%) from the MSM population were aimed outside the group, and approximately half of the flirts received by the MSM population were from non-MSM members. However, in some analyses we did include all members of the Nordic community and the results are generally consistent with results obtained using the more restrictive sample. Second, the time period, roughly two-months, was too short to study in detail the evolution of matched flirts over time. Last, the present results from the Qruiser.com community may reflect the particular sample and may not be generalizable to other populations.

We are currently analyzing the observed core structures in a broader context, and we plan to do a modeling study based on the findings in this study to address the question of the potential influence of these networks on disease spreading and intervention outcomes. In conclusion, we believe this study provides insights into the structure and dating patterns among MSM and that the results will be useful as baseline data in further modeling studies of such systems. Our findings of highly skewed contact patterns and one large core group consisting of homosexual men and men aged 31–40 years suggest that transmission through internet dating contact networks should be considered by public health authorities in future prevention interventions.

## Supporting Information

Figure S1 **The network of matched flirts involving MSM members by the end of the study period.** The network consists of 190 separate components; men are shown with black circles, while women are shown with white circles.
(DOC)

Figure S2 **Plots showing the probability that the MSM experienced a matched flirt during the study period as function of the core number and the mean/median number of contacts among the MSM during the study period as function of the core number.** A) The probability for MSM members to have a matched flirt as function of core number (MSM-MSM network); B) The mean and median number of contacts of MSM members as function of core (MSM-MSM network); C) The probability for MSM members to have a matched flirt as function of core numbers (MSM-Nordic network); D) The mean and median number of contacts experienced by MSM members as function of core number (MSM-Nordic network).
(DOC)

Table S1 **Probability for identical characteristics of MSM proposers and their MSM targets.**
(DOC)

Table S2 **Age associations between MSM proposers and their MSM flirt targets.**
(DOC)
