# Supplementary figures and images for: A Characterization of Internet Dating Network Structures among Nordic Men Who Have Sex with Men
Source: PLoS One. 2012 Jul 13;7(7):e39717. doi: 10.1371/journal.pone.0039717 (PMC3396616; doi:10.1371/journal.pone.0039717)

*
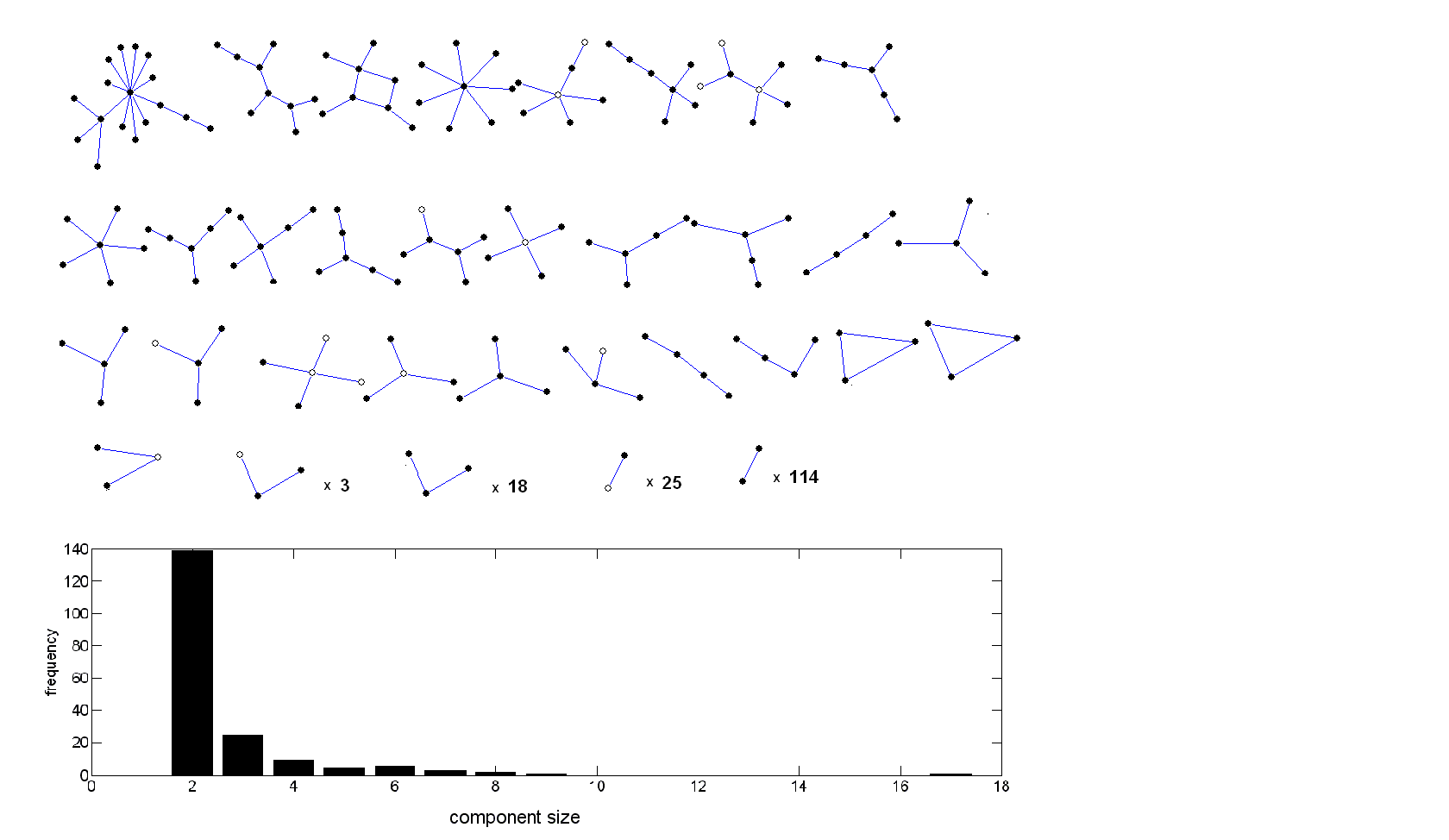
*

Supplement: Figure S1 — The network of matched flirts involving MSM members by the end of the study period. The network consists of 190 separate components; men are shown with black circles, while women are shown with white circles. (DOC) [file pone.0039717.s001.doc]

*
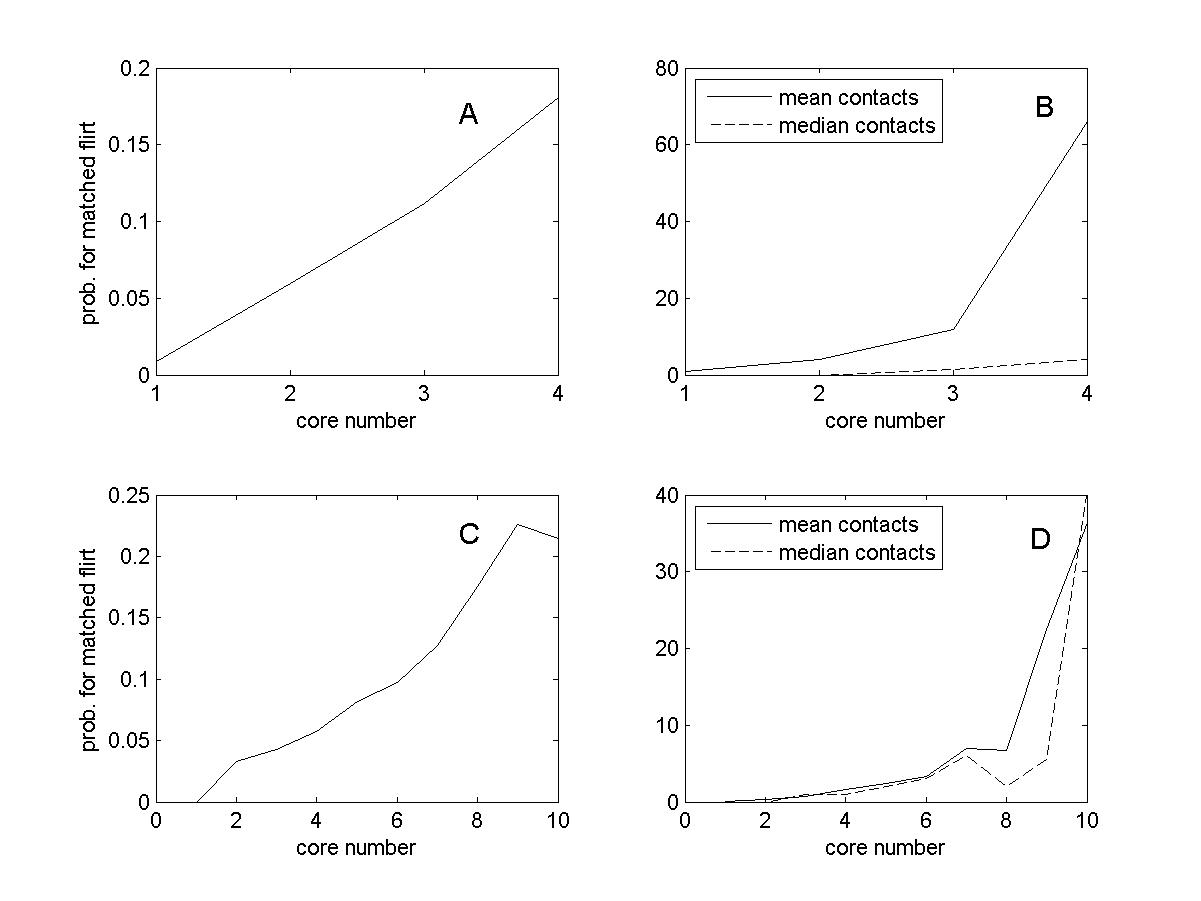
*

Supplement: Figure S2 — Plots showing the probability that the MSM experienced a matched flirt during the study period as function of the core number and the mean/median number of contacts among the MSM during the study period as function of the core number. A) The probability for MSM members to have a matched flirt as function of core number (MSM-MSM network); B) The mean and median number of contacts of MSM members as function of core (MSM-MSM network); C) The probability for MSM members to have a matched flirt as function of core numbers (MSM-Nordic network); D) The mean and median number of contacts experienced by MSM members as function of core number (MSM-Nordic network). (DOC) [file pone.0039717.s002.doc]
