# Supplementary material for: A Characterization of Internet Dating Network Structures among Nordic Men Who Have Sex with Men
Source: PLoS One. 2012 Jul 13;7(7):e39717. doi: 10.1371/journal.pone.0039717 (PMC3396616; doi:10.1371/journal.pone.0039717)
Supplement: Table S1 — Probability for identical characteristics of MSM proposers and their MSM targets. (DOC) [file pone.0039717.s003.doc]

|  | Probability | 95% CI |
| --- | --- | --- |
| Partner Preference  Sexual Style  Age  Civil Status | 0.21  0.30  0.30  0.64 | 0.10-0.24  0.28-0.33  0.29-0.31  0.63-0.65 |
| Webcam | 0.78 | 0.70-0.79 |
