# Supplementary material for: A Characterization of Internet Dating Network Structures among Nordic Men Who Have Sex with Men
Source: PLoS One. 2012 Jul 13;7(7):e39717. doi: 10.1371/journal.pone.0039717 (PMC3396616; doi:10.1371/journal.pone.0039717)
Supplement: Table S2 — Age associations between MSM proposers and their MSM flirt targets. (DOC) [file pone.0039717.s004.doc]

|  |  |  | Target |  |  |
| --- | --- | --- | --- | --- | --- |
| Proposer | Under20 | 20-29 | 30-39 | 40-49 | Over50 |
| Under 20 | 24.56 | 39.47 | 22.81 | 8.77 | 4.39 |
| 20-29 | 10.10 | 50.00 | 24.28 | 10.24 | 5.38 |
| 30-39 | 6.78 | 29.07 | 37.30 | 15.62 | 11.23 |
| 40-49 | 5.95 | 20.23 | 40.23 | 27.11 | 6.48 |
| Over 50 | 9.69 | 22.75 | 34.95 | 20.85 | 11.76 |

Numbers listed are probabilities that a proposer in age group (first column) sends flirts to MSM members in age group (upper row); row probabilities sum to one.
